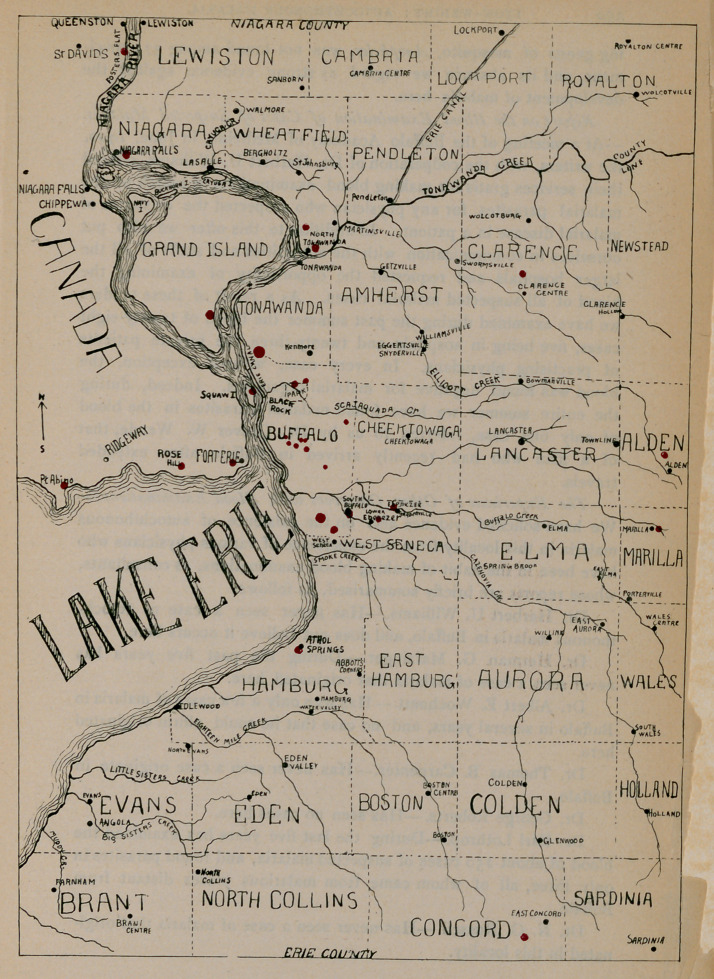# An Inquiry into the Existence of Autochthonous Malaria in Buffalo and Its Environs

**Published:** 1900-11

**Authors:** Irving P. Lyon, Alfred B. Wright

**Affiliations:** Buffalo, N.Y., 531 Franklin Street; Buffalo, N.Y., 531 Franklin Street


### AN INQUIRY INTO THE EXISTENCE OF AUTOCHTHONOUS MALARIA IN BUFFALO AND ITS ENVIRONS.

### PRELIMINARY REPORT ON SPECIES OF MOSQUITOES AND BLOOD EXAMINATIONS.

By IRVING P. LYON, M.D., and ALFRED B. WRIGHT, Stud. Med., Buffalo, N.Y.

[From the Pathological Laboratory of the University of Buffalo.]

THE investigations here recorded were suggested by two chief considerations, viz., first, the importance of accurately ascertaining the species of mosquitoes found in this locality with respect to establishing whether or not the *Anopheles* exists here, and, second, also, the frequence of the diagnosis of malaria by general practitioners of medicine.

A careful consideration of the first point seems to be one of the most feasible and scientific methods of throwing light on the second point, for it is now a demonstrated fact that various species of mosquitoes embraced within the genus *Anopheles* are the direct agents of the dissemination of malarial disease, and moreover the cumulative evidence of the more recent studies of this subject has focused strongly on the probability, that the *Anopheles* is the only active agent involved in the inoculation or production, by any means, of malaria.

We have, therefore, approached the investigation along two principal lines of inquiry, as follows: (1) What species of mosquitoes are found in and about Buffalo? (2) What evidence of the existence

1. Read before the Buffalo Academy of Medicine, October 16, 1900.

of autochthonous malaria here is furnished by examination of the blood?

Before presenting the facts which we have gleaned by pursuing these lines of inquiry, we wish to make clear that we recognise the occurrence of occasional cases of malaria here, in which the infection had been previously acquired at places far distant from Buffalo. We have, therefore, excluded from consideration all cases of malaria in persons who gave a history of recent previous malarial infection and in those who had traveled at a distance within a period of a few weeks previous to the development of malaria at this place. We have also excluded all malarial cases in which the history was not explicit and positive with respect to these points.

*Report on the Species of Mosquitoes collected in and about Buffalo.*—Our investigation of the species of mosquitoes found in this vicinity covers the period May 12—October 7, 1900. During this time we have examined 374 mosquitoes caught within and near the city of Buffalo, chiefly from points along Lake Erie and Niagara River. The infrequence of mosquitoes in this region and the difficulty of obtaining them, in spite of our unremitting efforts, explain why we have not a larger number of specimens upon which to base our report. For convenience of reference we have compiled the following table, showing the date and place of capture, and the number, species and sex of the mosquitoes.

TABLE OF OBSERVATIONS.

| Date of Capture. | Place. | Genus. | Species. | Sex. | No. |
|---|---|---|---|---|---|
| May 12, 1900 | *191 Barthel street | Culex | Stimulans | Female | 2 |
| " 13, " | Rose Hill, Canada | " | { " | " | 2 |
| | | | { Impiger | " | 1 |
| " 26, " | Foster's Flats, Can. (near whirlpool) | " | { Stimulans | " | 9 |
| | | | { Impiger | " | 5 |
| " 27, " | *22 Park street | " | Stimulans | " | 2 |
| " 27, " | Marilla, N. Y | " | " | " | 1 |
| " 29, " | Alden, N. Y | " | { " | " | 2 |
| | | | { Impiger | " | 2 |
| " 29, " | *Park Lake | " | Stimulans | " | 2 |
| " 30, " | *South Park, near lake | " | { " | " | 3 |
| | | | { Impiger | " | 5 |
| " 30, " | East Concord, N. Y., swamp | " | { " | " | 12 |
| | | | { Stimulans | " | 16 |
| June 2, 1900 | *221 North street | " | " | " | 1 |
| " 7, " | " " " | " | " | " | 1 |
| " 8, " | Gardenville, N. Y | " | { " | " | 3 |
| | | | { Impiger | " | 4 |
| " 9, " | Ebenezer, N. Y | " | { " | " | 3 |
| | | | { Stimulans | " | 1 |
| " 10, " | West Seneca, N. Y | " | " | " | 3 |
| " 15, " | Grand Island, N. Y | " | { " | " | 16 |
| | | | { Impiger | " | 25 |
| " 21, " | Clarence Center, N. Y | " | Stimulans | " | 1 |
| " 25, " | *221 North street | " | " | " | 1 |
| " 25, " | *Park Meadow | " | " | " | 3 |

| Date of Capture. | Place. | Genus. | Species. | Sex. | No. |
|---|---|---|---|---|---|
| June 28, 1900 | *100 High street | Culex | Impiger | Female | 1 |
| July 2, 1900 | *191 Barthel street | " | " | " | 1 |
| " 3, " | *221 North street | " | Stimulans | " | 1 |
| " 19, " | *O'Neil's Park | " | " | " | 1 |
| " 19, " | *O'Neil's Park, woods near | " | " | " | 9 |
| | | | Impiger | " | 14 |
| " 19 " | Point Abino, Canada | " | " | " | 23 |
| | | | Stimulans | " | 15 |
| " 22, " | *Squaw Island | " | " | " | 18 |
| | | | Impiger | " | 6 |
| " 23, " | Fort Erie, Canada, woods near | " | " | " | 17 |
| | | | Stimulans | " | 12 |
| August 4, 1900 | Athol Springs, N. Y | " | " | " | 5 |
| | | | " | Male | 4 |
| | | | Impiger | " | 12 |
| | | | " | Female | 5 |
| " 16, " | *22 Park street | " | Stimulans | " | 4 |
| " 16, " | *122 Grape street | " | " | " | 1 |
| " 19, " | 228 Gundary street, Tonawanda, N.Y. | " | " | " | 2 |
| | | | Impiger | " | 4 |
| September 2, 1900 | Tonawanda, N. Y., swamp | " | " | " | 27 |
| | | | Stimulans | " | 16 |
| " 6, " | *191 Barthel street | " | Impiger | " | 3 |
| " 8, " | *379 William street | " | " | " | 9 |
| | | | Stimulans | " | 6 |
| " 14, " | *191 Barthel street | " | " | " | 6 |
| | | | Impiger | " | 12 |
| " 16, " | *Cazenovia Park | " | Stimulans | " | 4 |
| " 25, " | Niagara Falls, N. Y | " | " | " | 1 |
| October 2, 1900 | *800 Main street | " | " | " | 1 |
| " 5, " | *77 High street | " | " | " | 3 |
| | | | †Pungens | Male | 1 |
| " 7, " | Athol Springs, N. Y | " | Stimulans | Female | 2 |
| | | | Impiger | " | 2 |

* Indicates points within the city of Buffalo.

† The wings of this specimen were injured so that the identification of its species, as *C. pungens*, by Dr. L. O. Howard, was qualified as "probable." As the *C. pungens* and C. *impiger* are very similar, this specimen may have been, perhaps, *C. impiger*, especially as no other specimen of *C. pungens* was found in our collection, whereas *C. impiger* constituted more than half of our specimens.

It will be seen by reference to this table of observations that the 374 mosquitoes were caught on thirty-nine different occasions, from thirty-one different places, of which fifteen were points within and sixteen points outside of the city of Buffalo. Many specimens were obtained from lowlands, marshes, ponds, and streams, chiefly in the region of Lake Erie and Niagara River, in fact from places to which public report attached a reputation of being malarious. The specimens were obtained by months as follows: May, 64; June, 63; July, 117; August, 37; September, 84; and October, 9. The observations were therefore made during six consecutive months, representing the "malaria season," and the distribution during these months was fairly uniform, with the exception of the last month, October. From points within the city, 121 mosquitoes were obtained, and 253 from places outside of Buffalo.

Of the 374 specimens, every mosquito belonged to the genus, *Culex*, and not a single example of *Anopheles*, the malaria-bearing variety, was found. The distribution by species and sex is shown as follows:

| | | | | |
|---|---|---|---|---|
| *Culex impiger* | 193 | Male . . 12 / Female . 181 | | |
| *Culex stimulans* | 180 | Male . . 4 / Female . 176 | Male . . 17 / Female . 357 | |
| *Culex pungens* | 1 | Male . . 1 | | |
| Total | 374 | 374 | 374 | |

It is thus shown that *C. impiger* and *C. stimulans* were about equally distributed, and that a third species, *C. pungens*, was represented by only a single specimen, the identification of which was only probable, due to the injury to its wings.

The unequal sex-distribution will also be noted. Only seventeen males, as opposed to 357 females, were found, and curiously also all of them, excepting one, *C. pungens*, at a single time and place. We are acquainted with no other statistics showing the sex distribution of adult mosquitoes in nature. The apparent rarity of males may find its explanation in the fact that males are not blood-suckers and, therefore, would not be so frequently found as females near people, where they would be caught.

In identifying the species we were assisted by Mr. E. P. Van Duzee, librarian of the Grosvenor Library, and by Dr. L. O. Howard, entomologist to the Division of Entomology, U. S. Department of Agriculture, Washington, D.C., to whom we wish to here record an expression of our appreciation and indebtedness for their very kind assistance. In collecting the mosquitoes we have received assistance from many interested and kind friends, of whom our especial thanks are due to Dr. and Mrs. DeLancey Rochester, Dr. Eugene A. Smith, Mr. Albert Frey, and Mr. T. F. Ellis. Our especial indebtedness and warmest thanks are due to Dr. Herbert U. Williams, professor of pathology, University of Buffalo, in whose laboratory the work was conducted, for his active coöperation and generous assistance for many months in this study.

To the courtesy of Mr. Charles A. Bentz, student of medicine in the University of Buffalo, we are indebted for the accompanying diagrammatic map of the region of Buffalo and its vicinity, from which we collected the mosquitoes for this study. The locations from which mosquitoes were caught are represented on the map by points in red.

In concluding the report of this part of our investigation, we may summarise the findings by the statement that the known malaria-bearing genus of mosquito, *Anopheles*, was not found among 374 specimens, and its absence we regard as strong evidence against the development of malaria here.

*Report on the Blood Examination of Cases of Suspected Malaria.*—At a meeting of the Buffalo Academy of Medicine, in June, 1900, the writers, with the coöperation of Dr. Herbert U. Williams, offered their services gratis for making blood examinations in search of the malarial parasites for any physician who suspected the existence of malarial disease in a patient. In addition to this offer we also put ourselves in communication with the authorities and staffs of all the larger hospitals and requested the opportunity of examining the blood of all suspected malarial cases. As a result of these tenders we have examined during the past summer the blood of twenty-eight cases, five being in hospitals and twenty-three the private patients of practising physicians. In every case, without exception, the blood was found negative for malarial organisms. Indeed, during the entire summer we have found malarial parasites in the blood of only one case, referred to us by Dr. Grover W. Wende, that of a man who had recently arrived in Buffalo after extended travels.

*The Experience of Other Observers with Blood Examinations.*—We have solicited evidence also on the existence of autochthonous malaria in this locality from the experience of various physicians who have been in the habit of making blood examinations, as consultants, whose reports are briefly summarised, as follows:

Dr. Herbert U. Williams.—Has never seen a case of autochthonous malaria in Buffalo, and does not believe it occurs here.

Dr. Herman G. Matzinger.—During the past five years has never seen a case of malaria that originated here.

Dr. Albert E. Woehnert.—Has seen only a few cases of malaria in Buffalo in several years, and no case that he could affirm originated here.

Dr. Thomas B. Carpenter.—Has never seen a case originate in Buffalo.

Dr. George Roberts.—Has seen no cases here.

Dr. Earl Lothrop.—During the last five years has examined the blood of about 250 cases of suspected malaria, and found parasites in only three, all of whom came from malarious places distant from Buffalo.

Dr. N. G. Russell.—Has never seen a case of malaria that originated in this locality.

Dr. Charles S. Jewett.—Is of the opinion that autochthonous malaria exists in the city; has found malarial parasites (tertian) in only one case which he thought acquired the infection in the city, but of this he is not certain, having made no special inquiries at the time of examination.

Dr. Julius Ullmann.—Has never seen a case originate in Buffalo.

Dr. Vertner Kenerson.—Has seen only one case that he suspected of local origin, but he made no special inquiries on this point.

We may add our own testimony to the above statements, to the effect that during the past two years one of us (Lyon) has made numerous blood examinations and has found malarial parasites in only four cases, all of whom acquired the infection at places far distant from Buffalo.

The unanimity of the evidence contained in the above statements against the autochthonous development of malarial infection in and about Buffalo—for many examinations were made, especially in the hospitals, on patients from contiguous towns—needs no comment.

We have also solicited the opinions of many physicians from all sections of Buffalo and from neighboring towns, who have not made blood examinations, as to the existence of malarial infection originating in this locality. The responses have varied in every degree from a positive affirmation to a flat denial of its origin here. Those who affirmed its occurrence offered no evidence in support of their opinion, save the clinical and therapeutic behavior of their cases. We may be permitted to comment in this connection that opinion, based alone on clinical and therapeutic observations, can claim only slight consideration as opposed to the scientific and demonstrable evidence of the microscope.

Inquiries at all the large hospitals as to the frequence of malarial cases, without reference to the source of origin of the infection, elicited the general reply that such cases were only rarely seen.

We may, therefore, summarise this part also of our inquiry with the statement that we have been unable to discover any evidence that proves the development of malaria in and about Buffalo in recent years.

Before closing this paper we wish to call attention to a few further points bearing on the subject.

*Geology and Topography.*—Our observations have been made in the city of Buffalo and in neighboring towns within a radius of twenty-five miles, the great majority of observations having been made in places close to Lake Erie or the Niagara River within or very close to the city of Buffalo. Buffalo is situated at the eastern end of Lake Erie, whose elevation above sea-level is 573 feet. The city rises above this level in general from 20 to 100 feet. The county of Erie and the surrounding district from which our observations were taken range from about 500 feet above sea-level on the north at Niagara Falls to about 1,500 feet in the hills in the southern part of Erie county. In general the district is underlain by a rock-bottom, composed chiefly of limestone or shale, and is covered with clay or clay and sand or gravel to a varying depth. The district is well drained by numerous streams and contains few swamps and lowlands. Niagara River, the outlet of Lake Erie, starting at Buffalo, flows northward through the district with a strong and rapid current. The district is only sparsely wooded, and is chiefly an agricultural country.

*Climate.*—The climate of the region is salubrious. The mortality records of Buffalo show one of the lowest death-rates recorded for any city in the United States. The prevailing winds are from the southwest, passing over Lake Erie before reaching Buffalo. The influence of the lake tends to produce an equable temperature at all seasons. The summer temperature is relatively low, the winter not severe. Humidity is increased by the influence of Lake Erie. The city and district are swept by frequent winds, blowing off the lake, sometimes of violent character. The weather of the past summer is shown by the United States Weather Bureau records to have been above the average in temperature, humidity and precipitation.

It is therefore seen that the geology, topography and climate of the region are all opposed to the expectation of finding malaria endemic here. The country is high, dry, cool, windy and sparsely wooded.

Special effort was made to study cases of suspected malaria and collect mosquitoes from places that are commonly called malarious, viz., Tonawanda,[1] Squaw Island, South Buffalo, and the swamps, ponds, and streams of the region, and many observations were made from these places, but always with negative results for the presence of malaria or the *Anopheles*.

It is well known that the *Anopheles* does not inhabit the interior of towns and is found in general only in rural districts near natural collections of water, such as ponds and streams, where it breeds. Thus, in notoriously malarious regions, such as the Roman Campagna and the Chesapeake Bay region, the *Anopheles* has not been found within the cities of these regions, viz., Rome and Baltimore, nor do

1. Further observations ought to be made especially at Tonawanda.

cases of malaria develop autochthonously within these cities, except sporadically. We did not therefore, a priori, expect to find *Anopheles* and autochthonous malaria within the city of Buffalo, and only, if at all, in the outside rural districts, and our special efforts were directed to obtaining evidence from outside places.

Inquiries at the Pan-American Exposition grounds, just outside of the park, where the soil has been upturned and artificial waterways have been in construction during the past summer, have shown that no cases of malaria have been reported among the workingmen employed there. We have several times attempted to find mosquitoes there, but always unsuccessfully.

The weather of the past summer has been more than usually favorable to the spread of mosquitoes. The average temperature, humidity and rainfall have been above the average. Still, mosquitoes have not been frequent and have been difficult to obtain, and the clinical diagnosis of malaria has probably not been more frequent than in previous years.

As previously stated, occasional cases of genuine malaria have been seen in Buffalo, imported from a distance. The return of our regiments from the South after the Spanish-American war, in 1898, brought back many cases of the disease. Had the *Anopheles* been a common inhabitant of this region, it is fair to assume that these imported cases of malaria would have afforded opportunity for the infection of the *Anopheles* and the subsequent development of an outbreak of malaria. This, however, did not occur. On the other hand, it is possible to suppose that the *Anopheles* might be imported to this region from a distance and become resident. In reply to the suggestion of this possibility, we need only say that we have failed to find it, and, too, in a season favorable to mosquitoes. Still we do not deny that *Anopheles* may have abounded here in the past or may do so in the future.

That mosquitoes, and even the *Anopheles*, may exceptionally be carried to a distance from their breeding-places is evidenced by the observation of Grassi, who observed a few specimens of *Anopheles* in a railway carriage traveling from Florence to Berlin. We have ourselves during the past week caught a mosquito (*Culex*) in a railway car on the Lehigh Valley Railroad, near Geneva, New York, in traveling from Buffalo. It may be that by such agencies and others the spread of *Anopheles* into new localities is effected, and in case the other conditions necessary are present, sporadic cases of malaria may arise. Such sporadic cases, due to such exceptional circumstances, would not however alter the general fact that malaria appears to be rare and is not endemic here. It seems to be a generally accepted fact that genuine malaria was in earlier years common throughout this region and through the northern and eastern states, although at present it is a rare condition away from the seacoast and in the country north of New Jersey.

The distance to which mosquitoes may be carried exceptionally by natural agencies is unknown. The available evidence tends strongly to show that mosquitoes usually remain close to their breeding-places and do not migrate far. They resist being carried by winds and storms and seek shelter against the violence of the elements. However, to what distance they may exceptionally be carried by violent storms is only conjectural. In this connection we may call attention to such a possibility from the cyclone that visited Buffalo last month. This storm traveled northward from the West Indies, passed Galveston, Texas, wrecking the city, and reached Buffalo on September 11th. The wind blew seventy-eight miles an hour and effected much damage. In the third and fourth weeks after the storm occurred, mosquitoes became more common in Buffalo than before, and much more frequent than is usual at this season of the year. Is it not possible that mosquitoes were brought by the cyclone and left in its track? Although we found no specimens of *Anopheles*, we have found since this storm a single specimen of *Culex pungens* (identification only probable), a species that we had not previously observed here.

We have had a secondary purpose in view in pursuing these investigations—namely, to contribute our minute share of evidence to the world-wide study that is now in progress of the relation of the distribution of the various species of mosquitoes to the production of malaria. All contributions, however, fragmentary, isolated and apparently insignificant, form an integral part of the foundation of evidence upon which the future knowledge of malaria-distribution must rest.

In conclusion we wish to state that we make no claim to having settled the question of the occurrence of autochthonous malaria here, but we believe that only by such studies as we have made can reliable data be obtained on which to base an opinion. It is sincerely hoped that further contributions to the subject will make certain what our work has made only very probable.

531 FRANKLIN STREET.

## Figures and Tables

**Figure f1:**